# MiR-20a-5p represses the multi-drug resistance of osteosarcoma by targeting the SDC2 gene

**DOI:** 10.1186/s12935-017-0470-2

**Published:** 2017-11-02

**Authors:** Fangfang Zhao, Youguang Pu, Mingda Cui, Haiyan Wang, Shanbao Cai

**Affiliations:** 1 Cancer Epigenetics Program, Anhui Cancer Hospital, West Branch of Anhui Provincial Hospital, Anhui Medical University, Hefei, 230031 Anhui China; 2 Xinxiang Medical University, Xinxiang, 453003 Henan China; 3 Department of Clinical Geriatrics, Anhui Provincial Hospital, Anhui Medical University, Hefei, 230031 Anhui China; 4 Department of Orthopedic Surgery and Cancer Epigenetics Program, Anhui Cancer Hospital, West Branch of Affiliated Provincial Hospital, Anhui Medical University, Hefei, 230031 Anhui China

**Keywords:** Osteosarcoma, Chemoresistance, miR-20a-5p, SDC2

## Abstract

**Background:**

As one of the hallmarks of cancer, chemoresistance hinders curative cancer chemotherapy in osteosarcoma (OS). MicroRNAs (miRNAs) act as key regulators of gene expression in diverse biological processes including the multi-chemoresistance of cancers.

**Methods:**

Based on the CCK8 experiments, we performed an RNA-seq-based miR-omic analysis of osteosarcoma (OS) cells (a multi-chemosensitive OS cell line G-292 and a multi-chemoresistant OS cell line SJSA-1) to detect the levels of miR-20a-5p. We predicted *Homo sapiens* syndecan 2 (SDC2) as one of the target genes of miR-20a-5p via several websites, which was further validated by detecting their expression of both mRNA and protein level in both the miR-20a-5p-mimic transfected G-292 and miR-20a-5p-antagomiR transfected SJSA-1 cells. The involvement of SDC2 with OS chemoresistance was checked by siRNA-mediated repression or overexpression of SDC2 gene. Cell viability was assessed by CCK8 assay.

**Results:**

We found that the miR-20a-5p level was higher in G-292 cells than in SJSA-1 cells. Forced expression of miR-20a-5p counteracted OS chemoresistance in both cell culture and tumor xenografts in nude mice. As one of miR-20a-5p’s targets, SDC2 was found to mediate the miR-20a-5p-induced repression of OS chemoresistance.

**Conclusions:**

Our results suggest that miR-20a-5p and SDC2 contribute to OS chemoresistance. The key players in the miR-20a-5p/SDC2 axis may be a potential diagnostic biomarker and therapeutic target for OS patients.

## Background

MiRNAs are a class of small non-coding regulatory RNA molecules that have been shown to be involved in a wide range of biological processes [1]. Their dysregulation has been associated with the development of diseases including cancer. The abnormal expression of miRNAs in cancer contributes to every aspect of tumor biology [2, 3], including drug resistance [4], which remains a major obstacle to effective treatment in patients [5]. As a hallmark of cancer, the multi-chemoresistance varies drastically among the cancer patients, the different cancer lesions or different regions of the same lesions within a single patient [6]. Despite years of intensive efforts, our knowledge of the cancer multi-chemoresistance remains very limited [7, 8]. To date, much effort has been exerted in analyzing the role of miRNAs in the development of chemoresistance in a variety of cancers. Several studies have shown that miRNA misregulation can increase chemoresistance in cancer cells if specific proteins are affected [9–11]. For instance, miR-33a was found to be upregulated in osteosarcoma (OS) chemoresistance and to promote resistance to cisplatin by downregulating TWIST [12].

As one of the well-studied miRNAs, miR-20a, a member of the miR-17-92 cluster, has been shown to function as an oncomir in many human cancers, including lung cancer [13], hepatocellular carcinoma [14], and gastric cancer [15]. In addition, miR-20, Rest and Wnt signaling is suggested to be involved in a regulatory circuit that can modulate the neural differentiation of neural progenitor cells [16]. It was also found that miR-20a induces cell radio-resistance by activating the PTEN/PI3K/Akt signaling pathway in hepatocellular carcinoma [17].

OS is the most common malignant primary bone tumor in children and adolescents [18, 19], and the mechanism for the OS chemoresistance remains largely unknown. In the present study, we performed a RNA-seq based-omic analysis for the differentially expressed genes in a multi-chemosensitive (G-292) versus a resistant (SJSA-1) OS cell lines. We showed here that miR-20a-5p represses the OS multi-chemoresistance via its down-regulation of the SDC2 gene, a newly identified target of miR-20a-5p.

## Materials and methods

### Cell lines and culture

Two osteosarcoma cell lines used in this study-G-292 (ATCC NO. CRL-1423) and SJSA-1 (ATCC NO. CRL-2098) were purchased from the ATCC (https://www.atcc.org/). The two cell lines were cultured in Dulbecco’s modified Eagle’s medium (Invitrogen, Carlsbad, CA, USA) supplemented with 10% fetal bovine serum (Invitrogen) and 1% glutamine at 37 °C in 5% CO_2_.

### The mimic/antagomiR/siRNA/overexpression plasmids transfection

All the mimic, antagomiR, siRNA, and the scramble sequence control (NC) as well as riboFECT CP transfection kits were supplied by Guangzhou Ribobio (Guangzhou, China). The mammalian expression constructs for SDC2 with GFP tag (EX-W2418-M98) were supplied by GeneCopoeia™ (http://www.genecopoeia.com/). Transfection of both ribonucleic acid reagents or plasmids mentioned above and the reporter plasmids in a Cignal Finder Pathway Reporter package (Qiagen, Hilden, Germany) was performed according to the manufacturer’s instruction.

The sequences used in this study are as follows:

si-SDC2:

5′-GAAACCACGACGCTGAATA-3′

hsa-miR-20a-5p

antagomiR: 5′-CUACCUGCACUAUAAGCACUUUA-3′

mimic:

sense 5′-UAAAGUGCUUAUAGUGCAGGUAG-3′

antisense 5′-CUACCUGCACUAUAAGCACUUUA-3′

### The luciferase reporter assay

Two partial sequences of the human SDC2 3′-untranslated region (276 bp, 1–276 and 533 bp, 1729–2261) with the miR-20a-5p targeting motif were cloned at the downstream of the firefly luciferase gene in pmiR-RB-REPORT™ to construct pmiR-RB-REPORT™-luc-SDC2-WT1 and pmiR-RB-REPORT™-luc-SDC2-WT2, respectively. Cells were seeded into 96-well plates at around 1 × 10^4^ cells per well and transfected with a mixture of 50 ng pmiR-RB-REPORT™-luc-SDC2-WT1/WT2, 5 ng Renilla plus 5 pmol mimic or scramble control (NC) nucleotides, with the riboFECT CP transfection reagents according to the manufacturer’s instruction. Both firefly and Renilla luciferase activities were measured 24 h after transfection by the Dual-Luciferase Reporter Assay System (Promega) using a Promega GloMax 20/20 luminometer. The relative firefly luciferase activities were normalized with the Renilla luciferase activities as a for transfection efficiency.

### Chemotherapeutics

Clinical grades of the following drugs were used (NCI dictionary of cancer terms, http://www.cancer.gov/dictionary), Dox (Haizheng, Zhejiang, China); Etop (Hengrui, Jiangsu, China); MTX (Lingnan, Guangdong, China) and CDDP (Haosen, Jiangsu, China) [5, 20, 21].

### Chemoresistance profiling (IC_50_ measurements)

Cells in the logarithmic phase of growth were seeded in triplicate in 96-well plates at the density of 0.5 × 10^4^/well and treated with fourfold serially diluted drugs for 72 h. Cell survival was then measured by a thiazolyl blue tetrazolium bromide (CCK8, 450 nm reading)-based cell proliferation assay [5]. Both the linear regression parameters and the IC_50_ (the concentration of drug required for 50% of cells to be killed) with the no-drug control as the reference were calculated. The relative chemoresistance was presented as the fold for each of the cell line over the lowest IC_50_ [22].

### Apoptosis analysis

Cells were harvested and rinsed with PBS twice. Then, 3 μl of FITC-labeled enhanced annexinV and 3 μl (20 μg/ml) of propidium iodide were added to 100 μl of cell suspension. After incubation in the dark for 15 min at room temperature, the samples were diluted with 100 μl of PBS. Flow cytometry was performed on a FACSCalibur instrument. The results were analyzed according to the manufacturer’s instructions. The experiments were performed independently three times, and a representative is shown.

### In vitro invasion assays

Cell invasion assays were performed in a 24-well plate with 8 mm pore size chamber inserts (Corning, USA). For invasion assays, 1 × 10^4^ cells stably expressing mimic, antagomiR or NC were placed into the upper chamber in each well with the matrigel-coated membrane, which was diluted in serum-free culture medium. In the assay, cells were suspended in 100 μl of DMEM without FBS when they were seeded into the upper chamber. In the lower chamber, 500 μl of DMEM supplemented with 10% FBS was added. After incubation for 30 h at 37 °C and 5% CO_2_, the membrane inserts were removed from the plate, and non-invading cells were removed from the upper surface of the membrane. Cells that moved to the bottom surface of the chamber were stained with 0.1% crystal violet for 30 min. The cells were then imaged and counted in at least five random fields using a CKX41 inverted microscope (Olympus, Tokyo, Japan). The assays were conducted three independent times [23].

### RNA analysis

RNA-seq analysis was performed by BGI-Tech of China, and RNA-seq library preparation and sequencing were performed by BGI (Shenzhen, China). Following purification, RNA was fragmented using divalent cations at an elevated temperature, and first-strand cDNA was synthesized using random hexamer primers and Superscript TMIII (Invitrogen™, Carlsbad, CA, USA). Second-strand cDNA was synthesized using buffer, dNTPs, RNaseH, and DNA polymerase I. Short fragments were purified with a QiaQuick PCR extraction kit (Qiagen) and resolved with EB buffer for end reparation and poly (A) addition. The short fragments were subsequently connected using sequencing adapters. After agarose gel electrophoresis, suitable fragments were used as templates for PCR amplification. During the QC steps, an Agilent 2100 Bioanaylzer and an ABI StepOnePlus Real-Time PCR System were used in quantification and qualification of the sample library. Finally, the library (200-bp insert) was sequenced using Illumina HiSeq2000 (Illumina Inc., San Diego, CA, USA). The single-end library was prepared following the protocol of the IlluminaTruSeq RNA Sample Preparation Kit (Illumina) [24].

Total RNA was isolated from the cells during the logarithmic phase using Trizol (Tiangen Biotech Co., Ltd., Beijing, China). For the mRNA analysis, the cDNA primed by oligo-dT was made with a prime Script RT reagent kit (Tiangen Biotech Co., Ltd., Beijing, China), and the mRNA level of SDC2 was quantified by a duplex-qRT-PCR analysis where the TaqMan probes with a different fluorescence for β-actin (provided by Shing Gene, Shanghai, China) were used in the FTC-3000P PCR instrument (Funglyn Biotech Inc., Canada). Using the 2^−ΔΔCt^ method, the normalization with the β-actin level was performed before the relative level of the target genes was compared. The sequences of primers and probes used for the qRT-PCR analysis are as follows:

HSDC2 F: 5′-CCTATTGATGACGATGACTACGC-3′

HSDC2 R: 5′-CCTATTGATGACGATGACTACGC-3′

HSDC2 probe: 5′-ROX-CCTATTGATGACGATGACTACGC-3′

hACTB F: 5′-GCCCATCTACGAGGGGTATG-3′

hACTB R: 5′-GAGGTAGTCAGTCAGGTCCCG-3′

hACTB probe: 5′-CY5-CCCCCATGCCATCCTGCGTC-3′

### Western blotting

Cells were lysed with a lysis buffer (60 mM Tris–HCl, pH 6.8, 2% SDS, 20% glycerol, 0.25% bromophenol blue, 1.25% 2-mercaptoethanol) and heated at 95 °C for 10 min before electrophoresis. The protein was separated by 12% SDS-PAGE and then transferred to polyvinyl difluoride membranes (Millipore, Bedford, MA) by electroblotting. After blocking with 5% non-fat dry milk, the blots were incubated with primary antibodies (against SDC2 and GAPDH). The target bands were revealed by an enhanced chemiluminescence reaction (Pierce), and the relative density (level) of proteins over the GAPDH band was quantified with the Gel-Pro Analyzer (Media Cybernetics). Anti-SDC2 (YT4490) was purchased from ImmunoWay (http://www.immunoway.com/index.asp) and anti-rabbit IgG (SA00001-2) was purchased from San Ying Biotechnology, China (https://www.ptglab.com/).

### In vivo studies

Animal experiments were performed as previously described [22]. Expressions of SDC2 protein were measured using immunochemical analysis on 5-mm slices of formalin fixed paraffin-embedded tumor xenografts in nude mice. Antigens were retrieved by pretreating dewaxed sections in a microwave oven at 750 W for 5 min in a citrate buffer (pH 6) processed with the Super Sensitive Link-Labeled Detection System (Biogenex, Menarini, Florence, Italy). The enzymatic activities were developed using 3-amino-9-ethylcarbazole (Dako, Milan, Italy) as a chromogenic substrate. Following counterstaining with Mayer hematoxylin (Invitrogen), slides were mounted in aqueous mounting medium (glycergel, Dako). Pictures were taken using a LEICA DM 4000B microscope, while the relative level of each protein was calculated using LEICA software, percentage of the mock over the chemotherapeutic treated tumors was calculated and plotted.

### Statistical analyses

The data are presented as the mean, and the error bars indicate the S.D. All statistical analyses were performed with GraphPad Prism 5. Two-tailed Student’s t test, a one-way analysis of variance or Mann–Whitney *U* test was used to calculate statistical significance. A *P* value of < 0.05 was considered significant.

## Results

### SDC2 is a positive regulator of the multi-chemoresistance of OS

Our previous result suggested that G-292 and SJSA-1 cell lines are the multi-chemosensitive and multi-chemoresistant OS cell lines, respectively [22]. To identify the mechanisms that govern the multi-chemoresistance of OS cells, we performed an RNA-seq-based miR-omic analysis of G-292 and SJSA-1 cells (GEO Accession Number: GSE89930). The results showed that a dozen of miRNAs were differentially expressed in the SJSA-1 and the G-292 cells and miR-20a-5p was selected as one of the studied target miRNAs. Here the expression of miR-20a-5p by miR-omic analysis was 10.50-fold higher in G-292 cells compared with SJSA-1 cells [25]. We further tested the level of miR-20a-5p in G-292 and SJSA-1 OS cell lines by qRT-PCR. The level of miR-20a-5p in G-292 was 8.27-fold higher than that in SJSA-1, indicating a higher expression of miR-20a-5p in the sensitive OS cells [25].

**Fig. 1:**
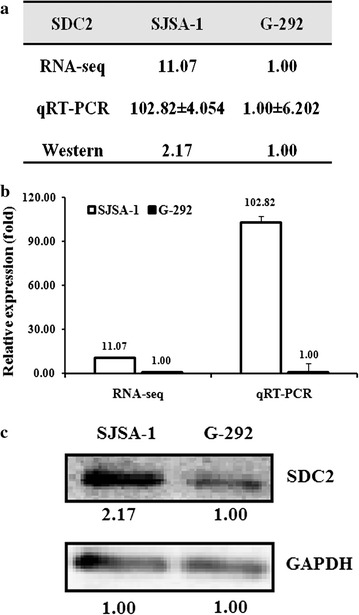
SDC2 is a negative regulator of OS multi-chemoresistance. The relative level (fold) of the SDC2 gene is also summarized in table (**a**), by miR-seq and qRT-PCR analyses in plot (**b**), analyzed by western analysis (**c**)

A given miRNA usually suppresses the expression of various target genes and thus regulates related pathways. We thus predicted the target genes of miR-20a-5p based on the following websites: TargetScan (http://www.targetscan.org/) and microRNA.org (http://www.microrna.org/microrna/getMirnaForm.do). We subsequently compared the expression pattern of shared predicted mRNAs between G-292 and SJSA-1 cells by the RNA-seq based miR-omic analysis (GEO Accession Number: GSE89930). Dozens of genes have been found that differentially expressed in the two cell lines. Among them, the SDC2 gene is one of the most significantly differentiated genes that negatively correlate with the expression of miR-20a-5p. Consequently, the expression level of SDC2 was higher in SJSA-1 than G-292 at both mRNA (RNA-seq based miR-omic: 11.07:1.00, and qRT-PCR analysis: 102.82:1.00) and protein level (western blot: 2.17:1.00) (Fig. 1a–c). The higher expression of SDC2 in multi-chemoresistant cells SJSA-1 suggests that SDC2 is a positive regulator of OS multi-drug resistance.

### The SDC2 gene is a direct target of miR-20a-5p in OS cells

**Fig. 2:**
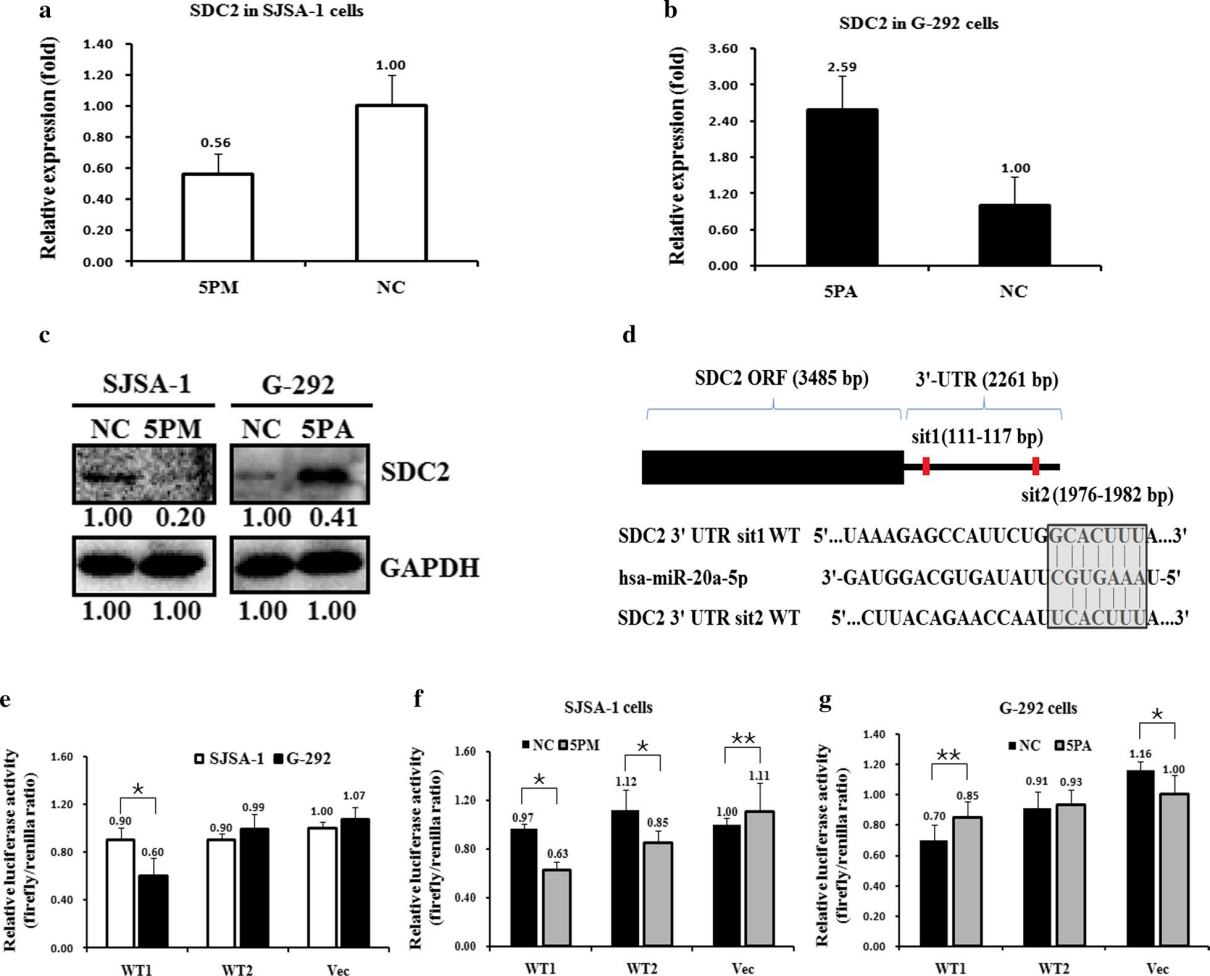
SDC2 is a target of miR-20a-5p in OS cells. The level of SDC2 mRNA (**a**, **b**) and protein (**c**) in the miR-20a-5p mimic (5PM)-transfected SJSA-1 cell and the miR-20a-5p antagomiR (5PA)-transfected G-292 cell versus the corresponding negative control (NC) determined by western analyses or qRT-PCR. **d** The sequences in the UTR region of the SDC2 gene targeted by miR-20a-5p (shaded part). **e**–**g** The relative luciferase activity (fold) of the reporter with wild-type (WT1 and WT2) SDC2-UTR or with no UTR (Vec) was determined in the miR-20a-5p mimic (in SJSA-1), antagomiR (in G-292) or corresponding mock-transfected OS cells. The Renilla luciferase activity of a co-transfected control plasmid was used to control the transfection efficiency. The representative results from three independent experiments are shown. Error bars represent the s.e.m. *P value < 0.05, **P value < 0.01 by Student’s *t* test

The miR-20a-5p level was significantly higher in G-292 cells than SJSA-1 cells. We found that SDC2 negatively correlates with the level of miR-20a-5p. To check whether SDC2 is one of the authentic targets of miR-20a-5p, we determined the SDC2 level in the miR-20a-5p mimic transfected SJSA-1 and the antagomiR transfected G-292 cells versus the NC (scramble sequence control) transfected. The transfection of miR-20a-5p mimic in SJSA-1 cells increased its expression to about 39-fold, whereas the transfection of miR-20a-5p antagomiR in G-292 cells significantly decreased its level to 19% [25]. Following the changes of the miR-20a-5p level, a miR-20a-5p mimic transfection brought down the SDC2 mRNA to 56% (Fig. 2a) and protein to nearly 20% (Fig. 2c) compared to that in the NC transfected SJSA-1 cells. As expected, miR-20a-5p antagomiR transfection increased the mRNA level of SDC2 by 2.59-folds (Fig. 2b) and the protein level by 3.78-folds in G-292 cells (Fig. 2c).

Sequence analysis revealed that 3′-UTR region of SDC2 contains two potential binding motifs for miR-20a-5p (termed sit1 and sit2, respectively) (Fig. 2d). To further conclude whether the SDC2 is a target of miR-20a-5p, the wild type 3′-UTR region of SDC2 was inserted downstream of the luciferase gene in the pmiR-RB-REPORT™-control vector to create pmiR-RB-REPORT™-SDC2 UTR WT1 and pmiR-RB-REPORT™-SDC2 UTR WT2 (Fig. 2e). The constructs pmiR-RB-REPORT™-SDC2 UTR WT and pmiR-RB-REPORT™ enhancer control were transfected into G-292 and SJSA-1 cells respectively, to determine whether the differentially expressed miR-20a-5p in OS cells of distinct chemoresistance is indeed functional. The pmiR-RB-REPORT™-SDC2-UTR WT1 gave the relative luciferase activities of 0.90 and 0.60 in SJSA-1 and G-292 cells, respectively, and the WT2 was 0.90 and 0.99 respectively (Fig. 2e). The transfection of miR-20a-5p-mimic into SJSA-1 cells significantly brought down the luciferase activity of pmiR-RB-REPORT™-SDC2-UTR WT constructs, whereas the control cells showed almost the same activity upon the transfection of miR-20a-5p-mimic (Fig. 2f). Meanwhile, the transfection of miR-34a-5p-antagomiR into G-292 cells raised the luciferase activity of pmiR-RB-REPORT™-SDC2-UTR WT constructs (Fig. 2g). It is worthy to notice that sit1 has a more profound effect against the changed level of miR-34a-5p in both G-292 and SJSA-1 cells, which indicated that miR-20a-5p binds more firmly to the sit1 UTR, compared to sit2 UTR. This is also in accordance with the sequence analysis that miR-20a-5p has seven base pairings for sit1 UTR whereas six base pairings for sit2 UTR (Fig. 2d). Getting together, SDC2 is indeed, a direct target of miR-20a-5p and may execute the miR-20a-5p’s repressing effect on the OS drug resistance.

### The SDC2 expression negatively correlates with the miR-20a-5p’s repressing effect on OS drug resistance

**Fig. 3:**
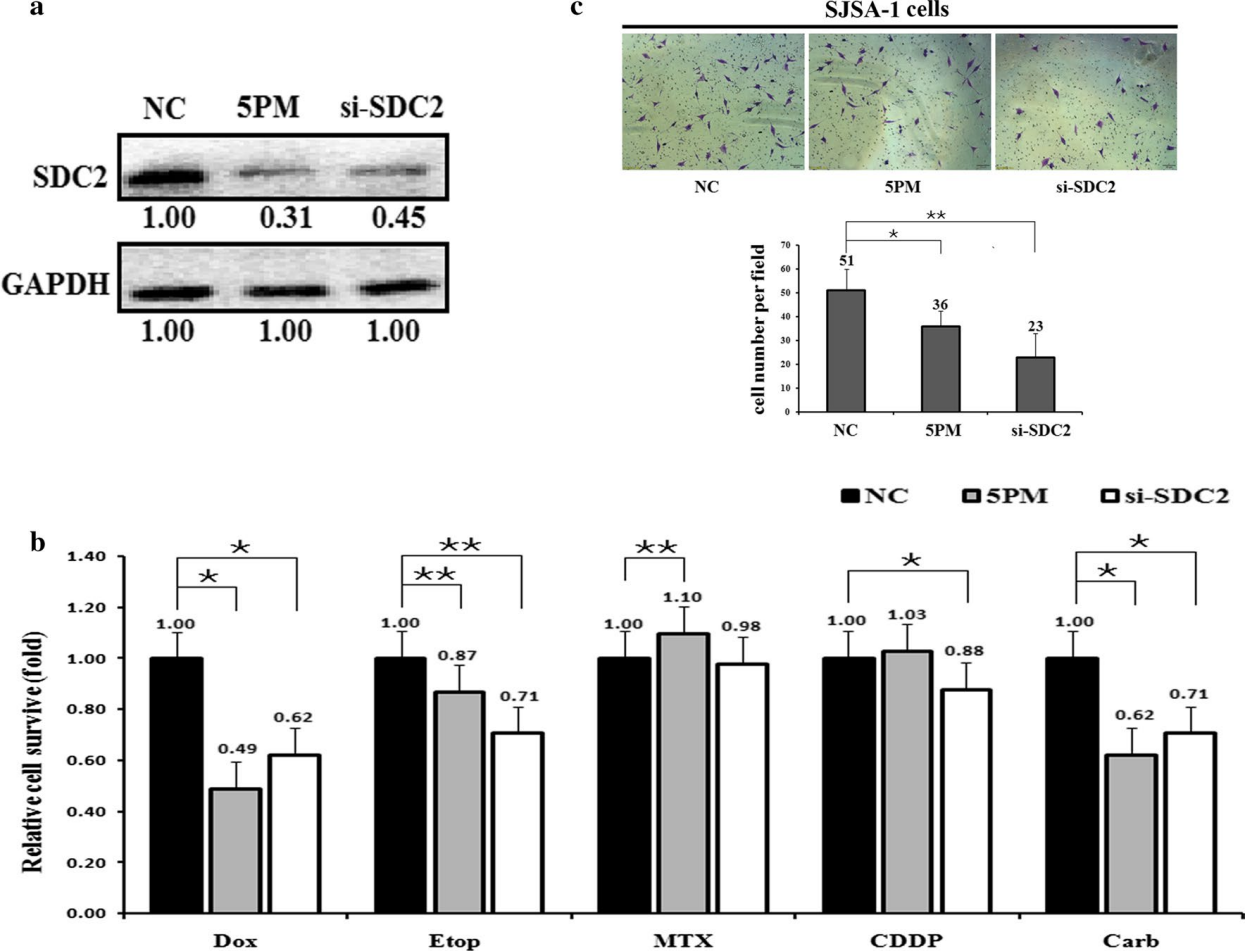
The effects of a forced reversal of the miR-20a-5p mimic (5PM) or si-SDC2 level on the chemoresistance of SJSA-1 cells. **a** The SDC2 protein level (western blot analysis) in the miR-20a-5p mimic or siRNA-transfected versus the NC-transfected SJSA-1 cells. **b** The IC_50_-dosed drug-induced cell death of SJSA-1 cells transfected with the miR-20a-5p mimic (5PM) or si-SDC2 versus the corresponding negative control (NC) assayed 72 h post-treatment. **c** MiR-20a-5p mimic or si-SDC2-transfected SJSA-1 cells showed lower invasion capacity compared with the NC-transfected. *P value < 0.05, **P value < 0.01 by Student’s *t* test

**Fig. 4:**
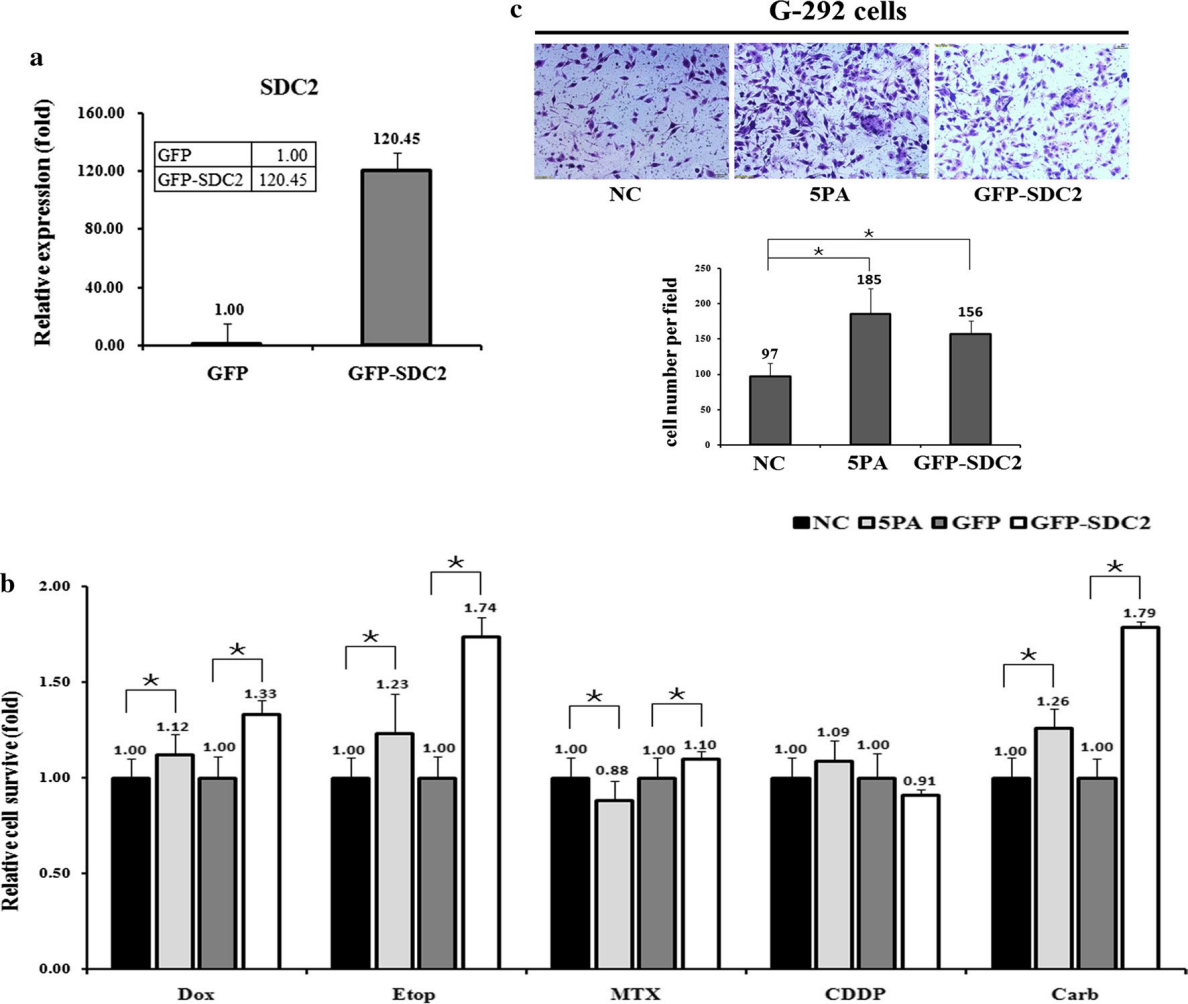
The effects of a forced reversal of the miR-20a-5p antagomiR (5PA) or overexpressed SDC2 level on the chemoresistance of G-292 cells. **a** The SDC2 protein level (western blot analysis) in the GFP-tagged overexpression construct-transfected versus the NC-transfected G-292 cells. **b** The IC_50_-dosed drug-induced cell death of G-292 cells transfected with the miR-20a-5p antagomiR (5PA) or the GFP-tagged overexpression construct versus the corresponding negative control (NC) assayed 72 h post-treatment. **c** MiR-20a-5p antagomiR (5PA) or overexpression construct-transfected G-292 cells showed higher invasion capacity then the NC-transfected. *P value < 0.05 by Student’s *t* test

To further demonstrate that miR-20a-5p modulates multi-drug resistance by repressing SDC2 expression in OS cells, we compared drug-triggered cell death in SJSA-1 cells transfected with miR-20a-5p mimic or SDC2 siRNA. The results showed that transfection with the miR-20a-5p mimic reduced the SDC2 level to 31% of that found in the NC transfected cells. In addition, SDC2 siRNA inhibited SDC2 protein expression to approximately 45% of the NC control (Fig. 3a). The transfection of miR-20a-5p mimic or si-SDC2 in SJSA-1 cells decreased the chemoresistance to some extent against the following five drugs: Dox, Etop, MTX, CDDP, Carb, except the mimic to MTX and CDDP (Fig. 3b). Consequently, the transfection of miR-20a-5p mimic or si-SDC2 in SJSA-1 cells showed lower invasion capacity compared to the control cells (Fig. 3c). Afterwards, we increased the level of SDC2 by transfection of miR-20a-5p antagomiR or overexpression of SDC2 in G-292 cells. In agreement with the elevated level of SDC2 (Fig. 4a), the cell survival rate was increased for all the five drugs, except the antagomiR to MTX and GFP-SDC2 to CDDP (Fig. 4b). This discrepancy suggested that SDC2 might not mediate OS chemoresistance in response to MTX and CDDP. Similarly, the invasion capacity was elevated with the transfection of either miR-20a-5p antagomiR or GFP-SDC2 in G-292 cells (Fig. 4c).

**Fig. 5:**
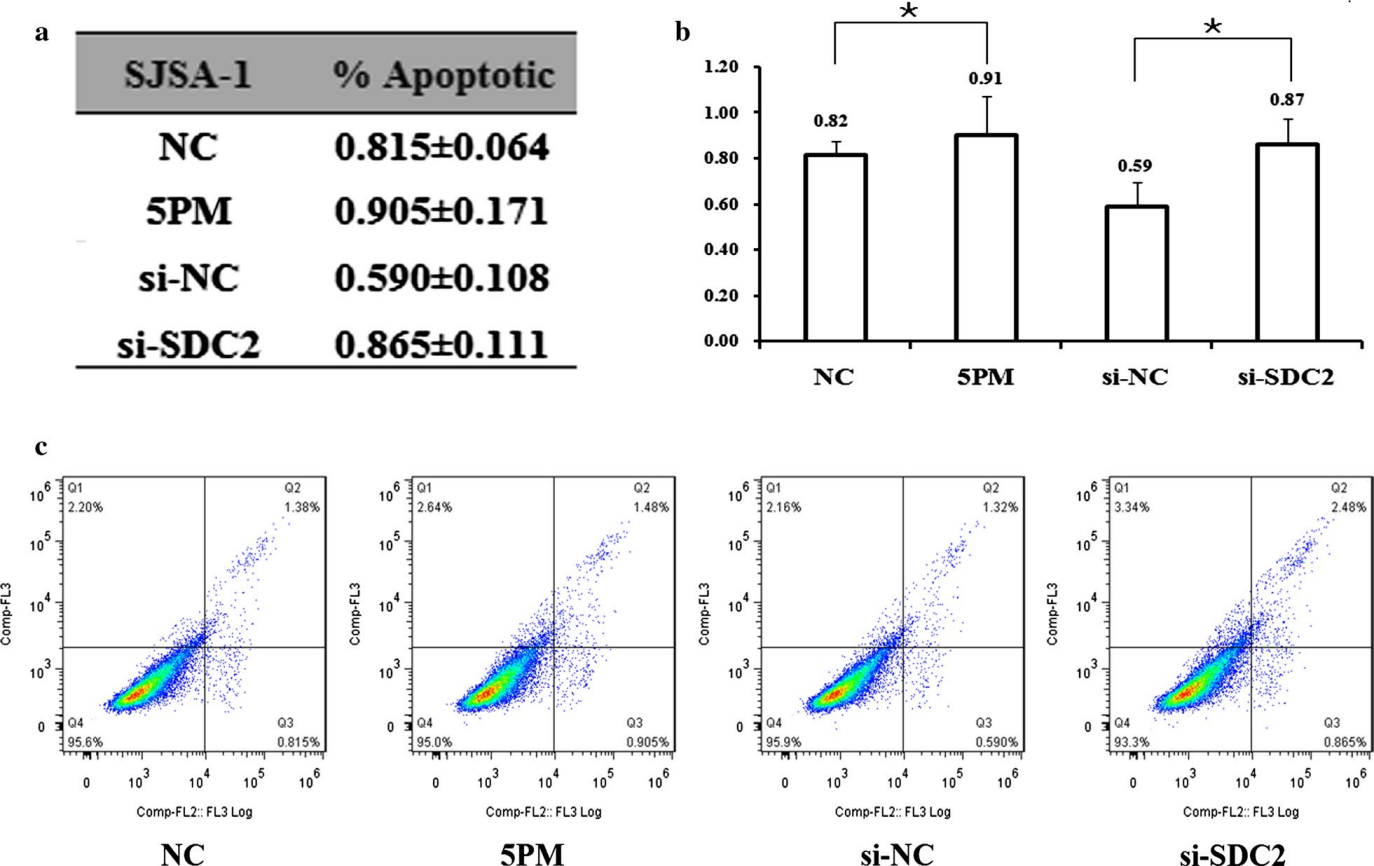
Effects of forced alteration of both miR-20a-5p and SDC2 levels on apoptosis in G-292 cells as determined by FACS analysis. *P value < 0.05 by Student’s *t* test

Additionally, the miR-20a-5p mimic mediated SDC2 downregulation in SJSA-1 cells and increased the percentage of apoptotic cells from 0.82 to 0.91%. Similarly, knockdown of SDC2 with siRNA also elevated the apoptotic ratio from 0.59 to 0.87% in SJSA-1 cells (Fig. 5a–c). This result indicated that low levels of miR-20a-5p promoted OS cell survival probably by increasing SDC2 expression. All of these observations suggest that SDC2 gene does contribute a great deal to the miR-20a-5p’s repressing effect on the OS drug resistance.

### MiR-20a-5p suppresses both growth and Dox drug resistance of the G-292 and SJSA-1-derived tumor xenografts in nude mice

**Fig. 6:**
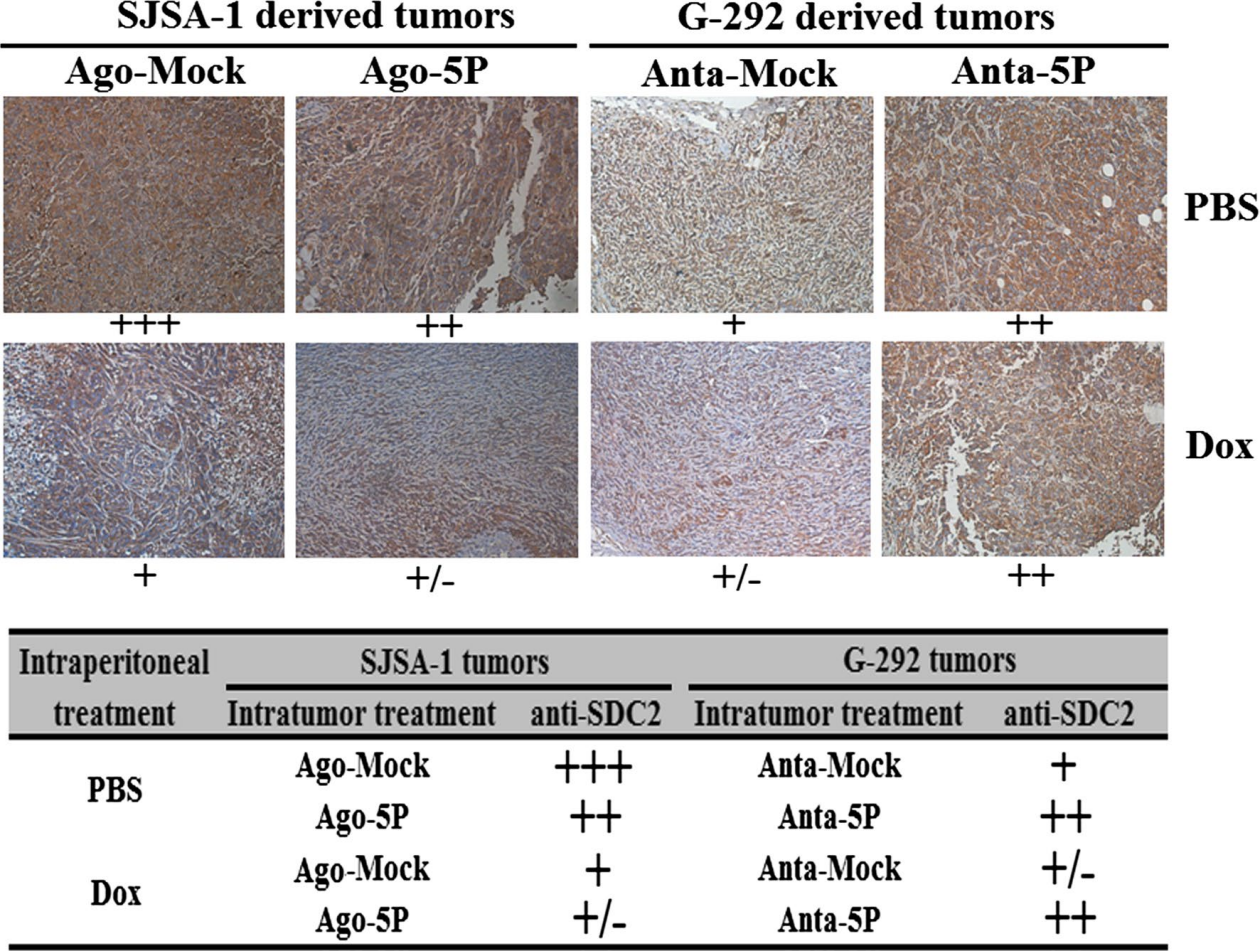
The SDC2 level in the miR-20a-5p agomiR-injected SJSA-1 and the miR-20a-5p antagomiR-injected G-292 tumor xenograft versus the NC-injected tumor xenografts determined by immunohistochemical staining. The SJSA-1 and G-292 tumor tissues from each group were fixed on one slide and immunostained for each indicated antibody. The levels of SDC2 protein in each group were determined by immunostaining and are summarized in the table (magnification: 200 ×)

Recently, miR-20a-5p was shown to suppress Dox chemoresistance of OS in tumor xenografts of nude mice via its repression of its target gene KIF26B [25]. In the present study, we semi-quantified via immuno-histological analysis the levels of SDC2 protein in the same set of the tumor tissues in mice that were subjected to an injection of Dox or PBS. The intratumoral injection of miR-20a-5p’s agomiR into SJSA-1 decreased SDC2 expression. By contrast, the injection of miR-20a-5p’s antagomiR into G-292 increased SDC2 expression in Dox- or PBS-treated mice (Fig. 6). The results further confirmed that miR-20a-5p has a profound negative effect on both the growth and chemoresistance of OS cell-derived tumor xenografts in nude mice.

## Discussion

OS chemoresistance is an important topic in the design of clinical treatment protocols because this resistance contributes to relapse and poor prognosis. In this study, we demonstrated that the expression level of miR-20a-5p was elevated in the chemosensitive OS cell line, suggesting that miR-20a-5p might participate in the regulation of OS chemoresistance. MiR-20a-5p expression has been shown to correlate with the development and progression of diverse cancer types [26–32]. For example, miR-20a-5p can be downregulated by glioblastoma hypoxia [31], which often promotes radioresistance and chemoresistance in cancer cells. However, knowledge of the contribution of miR-20a-5p to OS chemoresistance is still limited. In this investigation, we tested the impact of differential expression of miR-20a-5p on cell death in OS cells triggered by commonly used therapeutics.

To explore how miR-20a-5p affects chemoresistance regulation in OS, a luciferase reporter assay was performed to identify potential target genes of miR-20a-5p. The results showed that miR-20a-5p directly targeted *Homo sapiens* syndecan 2 (SDC2) in OS cells. SDC2, a cell surface heparan sulfate proteoglycan, is mainly expressed on mesenchymal cells and has been implicated in regulating a broad range of development and disease processes [33–35]. SDC2 exerts various functions in different cell types [36]. For example, SDC2 serves as a co-receptor for fibroblast growth factors and Wnt proteins and controls cell adhesion, proliferation, differentiation and apoptosis [37]. Of note, SDC2 contributes to osteosarcoma cell response to cytotoxic agents through interactions with Wnt/β-catenin signaling [38]. Apparently, SDC2 appears to have specific roles in bone cells but the studies of SDC2 effects with respect to tumor chemoresistance are rare. The current study is the first to demonstrate that SDC2 promotes the chemoresistance of OS cells via its upstream regulator miR-20a-5p. In this study, we also showed that SDC2 knockdown in OS cells promoted cell death. These data indicate that suppression of SDC2 inhibits cell survival and further implicate SDC2 as a potential therapeutic target for OS.

In summary, we demonstrated that a miR-20a-5p-centered axis dictates OS multi-chemoresistance. Because of its repressive effect on SDC2, decreasing miR-20a-5p expression promotes OS multi-drug resistance (at least for Dox, Etop and Carb, which were studied in this report) both in vitro and in vivo. Our data suggested that the miR-20a-5p level might serve as a potential biomarker of chemotherapy-resistant OS and that miR-20a-5p overexpression might aid in overcoming OS drug resistance.

## Conclusions

In conclusion, this study demonstrated that miR-20a-5p can regulate OS multi-drug resistance through its direct target gene SDC2 by targeting its 3′-UTR. Our findings suggest that miR-20a-5p may function as a potential candidate for preventing chemoresistance of OS, which may lead to additional new diagnostic and therapeutic approaches for OS and provide new insights into the posttranscriptional regulation of SDC2. Indeed, other regulators of SDC2 may also participate in OS chemoresistance, and our future studies should pay greater attention to examining how SDC2 is regulated in OS or other human tumors.

## Abbreviations

OS: osteosarcoma
MiR: microRNA
UTR: untranslated region
Dox: doxorubicin
Etop: etoposide
CDDP: cisplatin
MTX: methotrexate
SDC2: *Homo sapiens* syndecan 2
